# Trichobézoard gastrique: à propos d’un cas

**DOI:** 10.11604/pamj.2017.26.74.11826

**Published:** 2017-02-20

**Authors:** Maryame Ezziti, Fouad Haddad, Mohamed Tahiri, Wafaa Hliwa, Ahmed Bellabah, Wafaa Badre, Rabii Haddouch, Khalid El Hattbi, Mohamed Rachid Elfriyekh, Abdelaziz Fadil

**Affiliations:** 1 Service de Gastroentérologie, CHU Ibn Rochd, Casablanca, Maroc; 2 Service de Chirurgie Viscérale, CHU Ibn Rochd, Casablanca, Maroc

**Keywords:** Trichobézoard gastrique, fibroscopie oesogastroduodénale, gastrotomie, psychiatrie, Gastric trichobezoar, oesogastroduodenal fibroscopy, gastrotomy, psychiatry

## Abstract

Le trichobézoard est une affection rare, le plus souvent asymptomatique, mais de diagnostic facile par la fibroscopie œsogastroduodénale. Le traitement est le plus souvent chirurgical. On rapporte une observation d’un trichobézoard gastrique chez une fillette de 16 ans, qui a été extrait par gastrotomie, sans complications. Une prise en charge psychiatrique a été faite.

## Abstract (en)

Trichobezoar is rare, most often asymptomatic condition which can be easily diagnosed using oesogastroduodenal fibroscopy. Treatment is usually based on surgery. We here report the case of a 16-year old girl who underwent gastric trichobezoar extraction via gastrotomy, without complications. The patient even underwent psychiatric treatment.

## Introduction

Le trichobézoard gastrique est une affection rare désignant la présence inhabituelle de cheveux, sous forme de masse solide, au niveau de l’estomac. Le plus souvent asymptomatique, son diagnostic repose essentiellement sur la fibroscopie. Le traitement est souvent chirurgical [**1**]. Le but de ce travail est de discuter à travers d’un cas de trichobézoard gastrique les difficultés diagnostiques et les différentes méthodes thérapeutiques.

## Patient et observation

Il s’agit d’une fillette âgée de 16 ans sans antécédents particuliers qui consulte pour des douleurs abdominales de siège épigastrique à type de torsion sans autre signe associé. A l’interrogatoire la patiente est anxieuse avec la notion de trichophagie depuis une année. Cliniquement elle est pâle avec une sensibilité abdominale accentuée au niveau de l’épigastre.

**Figure 1:**
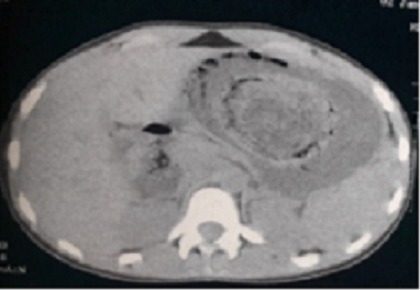
TDM abdominale en coupe axiale montrant un trichobézoard gastrique

**Figure 2:**
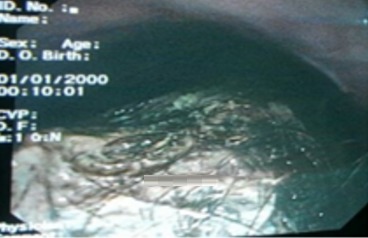
FOGD confirmant le trichobézoard

**Figure 3:**
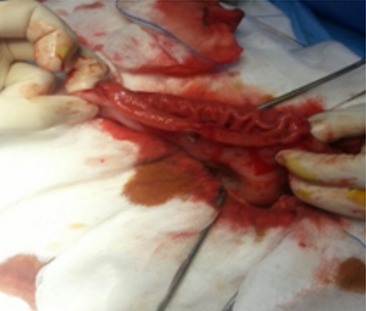
Gastrotomie longitudinale pour extraire le trichobézoard

**Figure 4:**
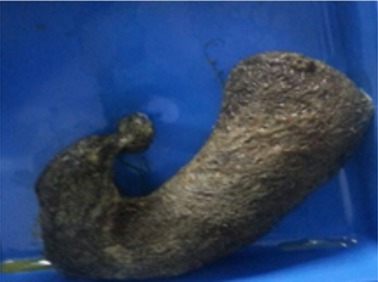
Énorme trichobézoard gastrique

A la tomodensitométrie (TDM) abdominale, il y a un contenu organisé dense piqueté occupant la lumière gastrique corporéo- antrale (Figure 1). La fibroscopie oeso-gastroduodénale (FOGD) a confirmé la présence d’un volumineux trichobézoard occupant la totalité de la cavité gastrique et une partie bulbaire qui a été déplacée par la pince à corps étranger au niveau de l’estomac, mais il était difficile à extraire (Figure 2). La malade a bénéficié d’une gastrotomie longitudinale avec extraction d’un volumineux trichobézoard mesurant 15 cm de grand axe (Figure 3, Figure 4). Les suites opératoires étaient simples. Une prise en charge psychiatrique a été effectuée.

## Discussion

Le trichobézoard est une affection rare, le sexe féminin est le plus touché (90% des cas) et l’âge de survenue est dans 80% des cas inférieur à 30 ans, avec un pic d’incidence entre 10 ans et 19 ans [1]. Des pathologies psychologiques sont parfois retrouvées comme un retard psychomoteur ou un isolement mais seulement 9% des enfants présentant un trichobézoard auraient de réels problèmes psychiatriques [2, 3]. Le trichobézoard est de siège gastrique le plus souvent mais il peut s’étendre à l’intestin grêle, voire au colon transverse, réalisant ainsi le syndrome de Rapunzel [4]. Chez notre patiente, il est de localisation gastrique et bulbaire. Le trichobézoard peut rester asymptomatique pendant longtemps ou se manifester par une gène épigastrique (80%), des douleurs abdominales (70%), des nausées ou vomissements (65%), une asthénie avec amaigrissement (38%) ou des troubles du transit (33%) à type de diarrhée ou de constipation [5–7].

Une complication peut être le mode de révélation de cette pathologie [7]. Il peut s’agir d’une hémorragie digestive haute due aux ulcérations pariétales, d’une occlusion mécanique gastrique ou grêlique [8, 9], d’une perforation gastrique ou grêlique avec péritonite ou abcès sous-phrénique [9–11], d’une fistule digestive [11, 12], d’une cholestase ou d’une pancréatite aiguë due à une obstruction de l’ampoule de Vater par un prolongement du trichobézoard (syndrome de Rapunzel) [13, 14]. À l’examen clinique, il existe dans 85% des cas, une masse abdominale bien limitée, lisse, ferme, mobile à localisation épigastrique. Une alopécie peut également être notée [5, 7]. Notre patiente ne présente pas de masse abdominale ni d’alopécie, elle présente une sensibilité abdominale.

Le diagnostic repose sur la FOGD qui reste l’examen de choix, en permettant la visualisation de cheveux enchevêtrés pathognomonique du trichobézoard. Elle peut, parfois avoir un intérêt thérapeutique en permettant l’extraction endoscopique de petits trichobézoards[7]. Cependant, à cause du volume du trichobézoard, cette extraction est dans la majorité des cas impossible, comme le cas de notre patiente et toute tentative comporte un risque de lésions œsophagiennes graves. Le cliché d’abdomen sans préparation peut montrer une masse arrondie dense ou hétérogène avec ou sans calcification se projetant sur l’aire gastrique [15]. L’échographie abdominale ne permet de poser le diagnostic que dans 25% des cas, en visualisant une bande superficielle, hyperéchogène, curviligne avec un net cône d’ombre postérieur [16, 17]. Le transit oesogastroduodénal objective une lacune intraluminale gastrique, mobile, à bords convexes, pouvant avoir une extension dans le duodénum [6]. Le transit du grêle complète l’exploration de l’intestin à la recherche d’une extension distale continue ou de fragments détachés [1]. Le scanner abdominal peut montrer une masse de volume variable, hétérogène, occupant presque la totalité de la lumière gastrique et constituée de multiples cercles concentriques de densités différentes réparties en bulbes d’oignon. Deux signes pathognomoniques et constants sont la présence de bulles d’air minuscules dispersées au sein de la masse et l’absence de toute attache de celle-ci à la paroi gastrique [15].

Plusieurs thérapeutiques ont été rapportées dans la littérature. Ainsi, en présence de trichobézoard de petite taille, certains auteurs proposent l’usage de boissons abondantes associées à la prise d’accélérateurs du transit, et d’autres une extraction endoscopique. D’autres auteurs proposent la fragmentation du trichobézoard, soit endoscopiquement par rayon laser et mini-explosion [18], soit par lithotripsie extracorporelle [19]. Outre un traitement incomplet, ces méthodes exposent à un risque de complications iatrogènes en particulier œsophagiennes ou d’occlusion intestinale sur fragment de trichobézoard. Le traitement est de ce fait souvent chirurgical. La chirurgie permet l’exploration de tout le tube digestif, l’extraction du trichobézoard gastrique à travers une gastrotomie, ainsi que l’extraction d’éventuels prolongements (queue) ou fragments bloqués à distance de l’estomac à travers une ou plusieurs entérotomies [1, 20]. Récemment, la voie laparoscopique a été proposée comme une alternative à la laparotomie [1]. Par ailleurs, une prise en charge psychiatrique doit souvent être instaurée chez les patients [1].

## Conclusion

Le trichobézoad est une pathologie rare, le diagnostic est confirmé par la fibroscopie oesogastroduodénale, l’exploration radiologique notamment par le scanner est primordiale, pour mettre en évidence d’autres localisations. Le traitement de choix est la chirurgie; cela ne doit pas occulter la prise en charge psychiatrique des patients.
